# Novel Insights Into the Epidemiological and Clinico-Pathological Profile of Triple-Negative Breast Cancer: Dissection of an Aggressive Variant

**DOI:** 10.7759/cureus.56124

**Published:** 2024-03-13

**Authors:** Vijay Sai Reddy M, Varsha Viswambharan, Varun Shetty, Sarthak Sharma

**Affiliations:** 1 General Surgery, Dr. D.Y. Patil Medical College Hospital and Research Center, Dr. D.Y. Patil Vidyapeeth, Pune, IND

**Keywords:** pathology, clinical, prognosis, staging, triple negative breast cancer

## Abstract

Objective

Triple-negative breast cancer (TNBC) represents an aggressive and prognostically poor variant of breast cancer. Over the years, detailed research has been conducted and published in Western literature. However, there lacks a detailed account of TNBC cases from the perspective of a low-volume institution. This study aims to assess the clinical features of TNBC, as well as their prognostic implications in a tertiary care centre.

Methods and materials

This prospective observational study took place at a tertiary health centre for two years, spanning from 2021 to 2023. The study aimed to investigate various clinicopathological and epidemiological parameters, thereby highlighting the shortcomings in the existing knowledge about the subject in the context of a low-volume centre, as well as additional contributing factors in developing countries like India. A group of 150 participants diagnosed with TNBC through biopsy and immunohistochemistry and >40 years of age were included in the study. Patients who tested positive for hormonal receptors and who refused to give consent for participation were excluded from the study. The study subjects were categorized according to their clinical TNM (cTNM) stage and eventually segregated into two primary heads, namely pre-surgery chemotherapy with breast-conserving surgery (BCS) after a good response, or modified radical mastectomy (MRM) upfront. Important demographic details, including age, socioeconomic status, and education, were also recorded. A comprehensive follow-up assessment post-treatment was performed to detect early recurrence. After data collection, the recurrence rates were correlated with the TNBC status to establish the aggressiveness of the cancer. Statistical analysis of the data was done using the Statistical Package for Social Sciences (SPSS) -16version software.

Results

The average age of the 150 participants in the study was 52.21 years (SD±4.89 years). The highest recorded age was 64 years, while the lowest recorded age was 45 years. In the study, it was observed that 41% of the participants diagnosed with TNBC had stage III disease, whereas 33.5% had stage I disease, 22% had stage IV disease and 3.6% had stage II disease. A total of 27.5% of individuals with TNBC exhibited metastases in various anatomical sites, whereas the other 72.5% did not show any signs of metastasis.

Conclusion

Triple-negative breast cancer has earned its position as a unique subtype of breast cancer due to its unusual molecular characteristics, aggressive behavior, limited treatment options, and poor prognosis. The lower per-capita income and limited knowledge pertaining to this variant, along with the absence of more specific treatment options, contribute to the already high levels of morbidity and mortality associated with this illness. To effectively address this unique and very virulent ailment and customize our strategies, it is imperative to do further comprehensive investigations, thereby enabling us to deliver the highest quality of medical attention to individuals afflicted by this pathology.

## Introduction

Breast cancer has become increasingly recognized as a heterogeneous illness in recent decades due to its varied presentation, large range of diagnostic tests, varying prognosis, and many treatment options [1]. Over time, numerous systems have been developed to classify carcinoma of the breast, of which immunohistochemistry has emerged as the most recent addition. In 2000, Perou et al. introduced a distinctive categorization scheme for breast cancer, which identified four distinct entities with varying prospects. Each subtype was found to possess distinct clinical characteristics and prognostic outcomes [2].

Triple-negative breast cancer (TNBC) constitutes only 5-20% of the total share of breast malignancies. It has demonstrated a higher level of aggressiveness in comparison to breast cancer which is positive for hormone receptors. During the initial 8-year period subsequent to diagnosis, around 33% of individuals diagnosed with TNBC may experience a distant recurrence [3]. Our study aims to provide insight into the epidemio-pathological characteristics of TNBC, which are poorly understood due to a lack of data relevant to TNBC cases in India.

## Materials and methods

**Figure 1:**
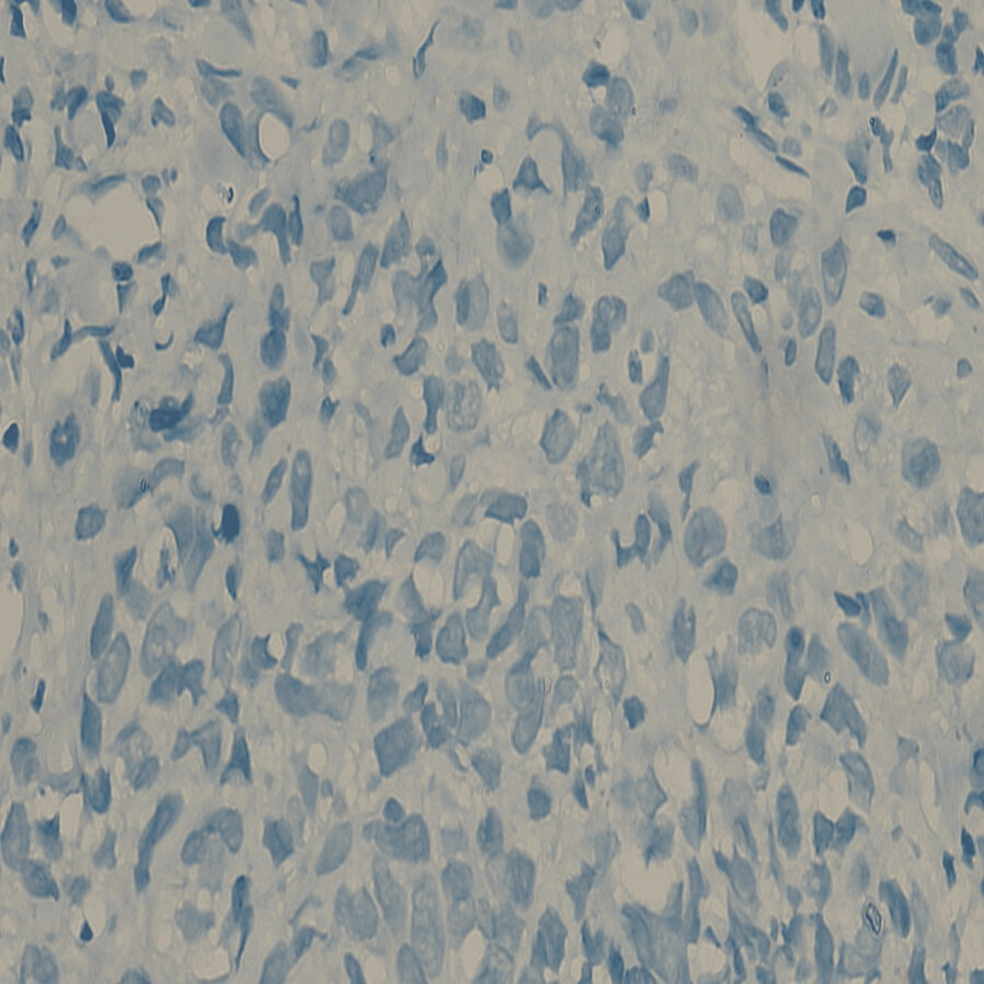
Immunohistochemical slide photograph showing Human Epidermal Growth Factor Receptor-2 (Her-2) receptor Negative status (Her-2-ve) in a case of triple-negative breast cancer (TNBC)

**Figure 2:**
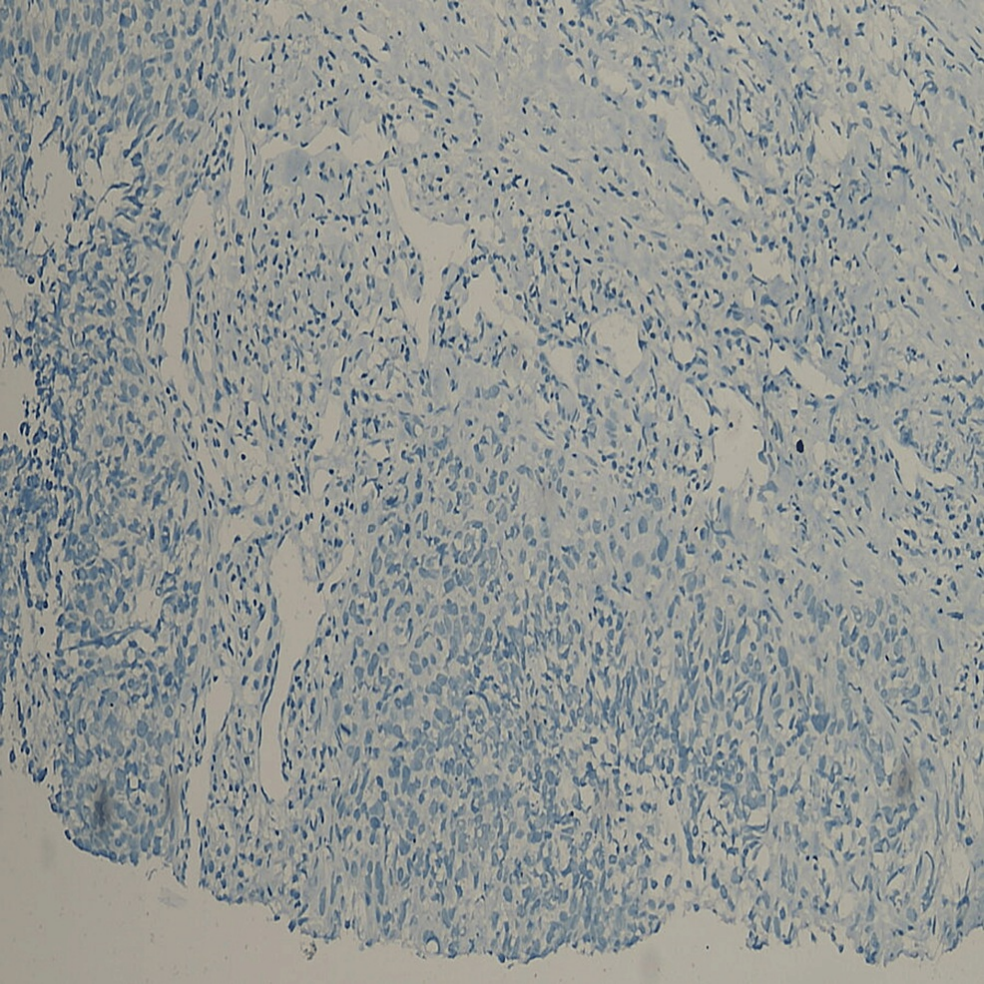
Immunohistochemical slide photograph showing Estrogen Receptor (ER-) and Progesterone receptor negative status (PR-) in a case of triple negative breast cancer (TNBC)

This prospective observational study took place at a tertiary health centre for a duration of two years, spanning from 2021 to 2023. A group of 150 participants with age >40 years diagnosed with TNBC confirmed by biopsy and immunohistochemistry (Figures 1-2 wherein triple negative hormone receptor status of one participant has been demonstrated) were included in the study. Important demographic details including age, socioeconomic status, and education were also recorded. Following the completion of the management, important pathological features of the tumor were recorded including the final histopathological staging, as well as the associated histological grading (Modified Bloom-Richardson grading). In accordance with pre-established protocol, subsequent to the completion of the surgical procedure, the patients were administered adjuvant chemotherapy and/or radiotherapy. The decision regarding the specific treatment modality was determined based on the final histopathological grading of the tumor as well as its overall aggressiveness. A comprehensive follow-up assessment post-treatment was performed to detect early recurrence. After data collection, the recurrence rates were correlated with the TNBC status to establish the aggressiveness of the cancer. The statistical analysis of the data was done using the SPSS-16 version software (SPSS Inc. Released 2007. SPSS for Windows, Version 16.0. Chicago, SPSS Inc.).

## Results

The overall age of the participant population was 52.21 years (SD ±4.89), of which the oldest and youngest participants were aged 64 and 45, respectively. Of the sample population, 35% were between the ages of 56 and 60. Based on the Modified Kuppu Swami Scale, 36% of TNBC patients are classified as upper-lower class, while 27.5% are classified as lower-middle class. Thirty percent of the participant cohort were illiterate. 

In the study, it was shown that 41% of the participants with TNBC had stage III disease, whereas 33.5% had stage I disease, 22% had stage IV disease and 3.6% had stage II disease. Among the participants with TNBC, 27.5% had metastases in various anatomical sites, whereas the remaining 72.5% did not display any signs of metastatic lesions.

Among the individuals with this receptor-deficient variant, the most prevalent histological subtype was invasive ductal carcinoma, accounting for 76% of cases as shown in Table 1. This was followed by invasive lobular carcinoma, which constituted 18% of cases, and invasive papillary carcinoma, which was observed in 2.66% of subjects. In the study cohort, 21% of individuals diagnosed with TNBC exhibited lymph node involvement, while the remaining 79% did not present with such involvement.

**Table 1:** Histopathological subtypes of study participants

Histopathological Type	Frequency (n)	Percent (%)
Invasive Ductal	114	76.0
Invasive Lobular	27	18.0
Invasive Papillary	4	2.66
Medullary	1	0.68
Mucinous	4	2.66

A total of 72% of individuals diagnosed with TNBC underwent treatment with modified radical mastectomy in combination with chemotherapy, while the remaining 28% of subjects received neoadjuvant chemotherapy. A total of 27% of participants diagnosed with TNBC experienced recurrence during a two-year period following therapy, with the remaining 73% achieving complete remission.

## Discussion

TNBC constitutes a significant variant of breast malignancy in terms of its aggressive course, pathological complexity, and diversity in clinical presentation. The pathophysiology of the disease exhibits a spectrum of manifestations, ranging from a mild presentation to a severe state with a heightened propensity for recurrence, contingent upon the gravity of the ailment [4].

One of the most prominent challenges encountered in the management of this variant is the widespread absence of targeted agents against hormonal receptors, especially for the general population. In recent years, there have been notable advancements in the development of therapies targeting these three receptors, which have demonstrated considerable potential as prospective treatments for breast cancer. The fundamental challenge in treating TNBC is the ongoing evaluation of targeted chemotherapeutic drugs and their limited accessibility to the middle-income population. The present investigation involved a sample of 150 patients, whose average age was determined to be 52.21 years (standard deviation ±4.89).

The ages of the participants ranged from 45 to 64 years, respectively. The obtained outcome was similar to the findings reported by Thike et al. and Rao et al. in their respective studies [5,6]. This variation in the onset may be attributed to the limited study participants. According to a study by Yang et al., there was a significant difference in the age at which menarche occurred between those with TNBC and those with other types of breast cancer, which is consistent with the findings of our study. Based on our research findings, it was determined that the majority of cases had Stage III TNBC on presentation [7].

These aforementioned observations on the prevalence of advanced stages at the time of diagnosis are likely influenced by low socioeconomic levels and limited access to advanced healthcare in rural areas of India. Consequently, these factors may contribute to the unfavorable 5-year survival rates observed in patients with TNBC. In the performed research, it was shown that 27% of patients diagnosed with TNBC experienced recurrence within a two-year period following therapy, while the remaining 73% achieved complete remission. In Sarin R et al.'s study, the observed recurrence rate was comparatively low, at 11.7% among the patients [8,9].

However, in a comparable study conducted by Zhang et al., the recurrence rate was notably higher at 19%. The high rates of recurrence observed in this context cannot be solely ascribed to a singular cause, as there are multiple factors that contribute to this phenomenon. These factors include insufficient investment in research and development in developing nations, resulting in a delay in the provision of targeted medications, particularly at affordable prices [10].

The current research was subject to certain limitations, including a relatively small sample size and a limited duration of follow-up. Nevertheless, our research aims to extend its scope by including a larger sample population, thereby yielding more precise and reliable data that can better reflect the characteristics of the intended population.

## Conclusions

TNBC has earned its position as a unique subtype of breast cancer due to its aggressive behavior, limited treatment options, and poor prognosis. The lower per-capita income and limited knowledge about this variant, along with the absence of more specific treatment options, contribute to the already high levels of morbidity and mortality associated with this illness. Lower socio-economic status and illiteracy are additional contributors to the delayed presentation and subsequently, have an impact on prognosis, especially in developing countries like India. To effectively address this unique and very virulent ailment and customize our strategies, it is imperative to do further comprehensive investigations thereby enabling us to deliver the highest quality of medical attention to individuals afflicted by this pathology.
